# Amine‐Functionalized Activated Carbon Monoliths by 3D Printing for Direct Air Capture

**DOI:** 10.1002/gch2.70105

**Published:** 2026-04-10

**Authors:** Johanna Fricke, Zoltán Bacsik, Christine Schütz, Marc Rüggeberg, Anju Pal, Niklas Hedin, Jiayin Yuan

**Affiliations:** ^1^ Department of Chemistry Stockholm University Stockholm Sweden; ^2^ Volkswagen AG, Group Innovation Wolfsburg Germany; ^3^ Institute of Chemistry University of Miskolc Miskolc Hungary

**Keywords:** activated carbon, CO_2_ adsorption, direct air capture, direct ink writing, graphene oxide, nanoporous

## Abstract

Direct air capture (DAC) is an emerging technology that supports mitigating climate change. However, its large‐scale deployment is hindered by high energy demands and material costs. In this study, we present a novel porous sorbent material for DAC using 3D‐printed activated carbon monoliths functionalized with an aminosilane compound. The monoliths were fabricated via direct ink writing and subsequently modified with 3‐aminopropyltriethoxysilane (APTES) to introduce chemisorption sites for CO_2_. Structural and chemical analyses confirmed successful grafting of amines without compromising the monolithic architecture. The resulting monoliths demonstrated enhanced CO_2_ uptake at atmospheric concentrations (0.25 mmol g^−1^ at 0.04 kPa). IR spectroscopy revealed that the functionalized monoliths chemisorb CO_2_ from ambient air as ammonium carbamate. Chemisorbed CO_2_ can be desorbed at a temperature of 75°C, indicating a low energy requirement for a DAC process. To the best of our knowledge, this paper is the first published application of aminosilane‐functionalized activated carbon for DAC, highlighting its potential as a cost‐effective and scalable sorbent material.

## Graphical abstract

Amine‐functionalized, 3D‐printed activated carbon monoliths enable efficient CO_2_ capture from ambient air. The tailored architecture improves heat and mass transfer, while chemisorption sites ensure high uptake at low concentrations. Regeneration at only 75°C highlights the potential for energy‐efficient direct air capture (DAC) using scalable, customizable carbon‐based structures.

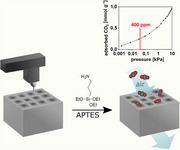

## 1 Introduction

The increasing concentration of carbon dioxide in the atmosphere challenges the stability of the global climate [1], and a mix of actions and technologies is needed to meet the 1.5°C warming target of the Paris accord. Among these, negative emission technologies (NETs), which actively remove CO_2_ from the atmosphere, complement emission reduction measures [2]. One particularly promising NET is direct air capture (DAC), which includes techniques for directly extracting CO_2_ from ambient air, for example, by adsorption. DAC can be deployed site‐independently as long as there is cost‐effective renewable energy available. It requires significantly less land use than other NETs, such as afforestation [3]. Challenges are in the scale of removal, as it needs to be able to remove gigatons of CO_2_ to have a significant impact on the global mean temperature and remain compatible with the 1.5°C target of the Paris accord [2], and in the currently high energy duty [4]. Therefore, further research and engineering is key to realize widespread deployment.

DAC can be performed by different techniques, enabling the extraction of CO_2_ from ambient air. Commonly, temperature swing adsorption (TSA) processes are studied and explored. In these cyclic and semi‐batch processes, CO_2_ is selectively captured from ambient air in the adsorption step and released as a concentrated stream in the desorption step by applying heat [5]. At present, the cost per ton of CO_2_ captured by DAC is too high to allow for large‐scale deployment, and one of the main cost drivers for DAC is the high energy demand during the desorption step [6]. The energy demand associated with desorption can be reduced by optimizing the process configuration and by improving the properties of the structured adsorbent.

In each cycle, the sorbent material must be heated to a sufficiently high temperature to enable the release of the captured CO_2_. Depending on the sorbent material used, desorption temperatures typically range from 80°C to 150°C [7]. However, most commonly used sorbents of today are typically having low thermal conductivities, which in turn renders the associated TSA slow.

The most currently studied adsorbents for DAC are, e.g., porous silica, zeolites, metal organic frameworks (MOFs), and polymers. These sorbents have a poor thermal conductivity of typically <0.5 W m^−1^ K^−1^ [8], which makes the installations excessively large and the cost per ton of CO_2_ captured high, if special measures are not taken [9, 10]. Developing sorbents with sufficiently high thermal conductivity improves heat transfer and decreases the energy use during the regeneration phase, supporting the process efficiency and scalability of DAC [8]. Such sorbents are needed in rapid TSA (RTSA) processes [11] to reduce the capital expenses and the size of the installations. In this context, activated carbon is a promising candidate, as it is an inexpensive, abundant, and non‐toxic material with a high thermal conductivity compared to the above‐mentioned sorbent materials for DAC [8]. However, activated carbon is a physisorbent for CO_2_, which shows a low adsorption capacity and selectivity for CO_2_ under atmospheric CO_2_ concentration [5]. By modifying the surface with amines, chemisorption can be enabled, which improves the CO_2_ adsorption [12, 13]. A well‐established strategy to introduce amines is the use of aminosilane compounds, which can form covalent bonds to functional oxygenated groups on the surface of a substrate [13].

Another common approach is the physical impregnation of amine‐bearing compounds into porous substrates [12, 13]. Several amine‐impregnated carbon‐based sorbents have been reported to exhibit high CO_2_ uptakes under DAC‐relevant conditions (see Table S1). For example, Song et al. demonstrated that a polyethyleneimine‐impregnated carbon aerogel can achieve a CO_2_ uptake of approximately 0.9 mmol g^−1^ at 400 ppm CO_2_ under dry conditions at 25°C [14]. However, impregnated amines are prone to volatilization and migration, which limits their long‐term stability. Although covalent grafting of amines onto carbon supports could improve thermal stability and long‐term durability [12, 13], this strategy has not yet been explored for DAC, including the use of aminosilane‐modified activated carbon. However, there have been previous studies presenting aminosilane‐functionalized activated carbon for CO_2_ adsorption at concentrations larger than 400 ppm. Wang Seog et al. [15] functionalized activated carbon with 3‐aminopropyltriethoxysilane (APTES) and examined the CO_2_ adsorption capacity via thermogravimetric analysis. The APTES‐functionalized carbon showed an uptake of ca. 0.98 mmol g^−1^ at 1 bar of CO_2_ and 25°C, which was lower than the uptake of 1.25 mmol g^−1^ of the activated carbon prior to functionalization. Lu et al. [16] grafted APTES on granular activated carbon, which improved the CO_2_ uptake of the activated carbon slightly from 0.57 to 0.60 mmol g^−1^ at a CO_2_ concentration of 10% at 1 bar in dry air. Saaroni et al. [17] showed significant improvement of CO_2_ adsorption in dynamic measurements with pure CO_2_ after APTES‐functionalization of activated carbon.

State‐of‐the‐art sorbents are mostly shaped into granules or pellets, and for desorption, external heating is applied in packed beds. This approach leads to long heating times due to poor heat transfer [18, 19, 20]. Granules and pellets are thus not suitable for RTSA processes, and instead, forms such as monoliths and fibers are needed for enabling short cycles [21, 22]. More specifically, shaping the sorbents into monolithic structures can increase the process efficiency compared to granules by reducing the pressure drop and improving heat and mass transfer [20, 21, 22, 23, 24], and it is also possible to assure effective heat integration using monoliths for RTSA. (US Pat. No. 8444750)

Using 3D printing approaches for the creation of monoliths provides flexibility and variety in shape and size of structures, making it possible to design tailored structures for specific use cases [25]. Direct ink writing is a 3D printing technique in which a shear‐thinning gel with appropriate properties is extruded layer‐wise through a nozzle to create the desired 3D shape [23, 26].

In this study, an activated carbon monolith was 3D printed by direct ink writing, and an aminosilane‐functionalization was applied to the monolith. The surface of the monolith was grafted uniformly with aminosilane without damaging the printed structure. The modified monolith was then tested for CO_2_ adsorption at atmospheric concentrations to assess its potential for DAC, showing that the material adsorbed CO_2_ efficiently through chemisorption at low CO_2_ levels, with desorption occurring at temperatures between 35°C and 75°C.

## 2 Results and Discussion

The aminated and 3D printed carbon monoliths were designed to improve the mass and heat transfer during the desorption step, reducing the energy penalty for DAC. The following results are discussed in terms of successful fabrication and CO_2_ adsorption and desorption characteristics.

### 2.1 3D Printed Carbon Monoliths

**FIGURE 1:**
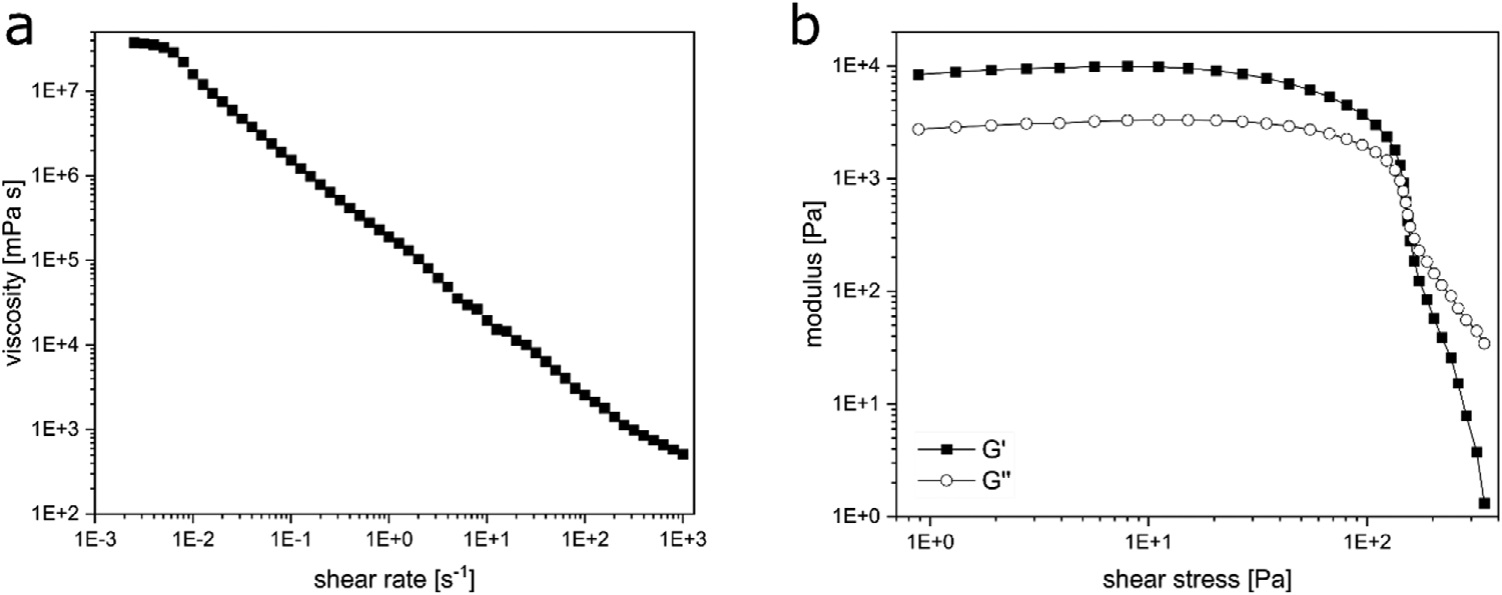
Flow curve (a) and controlled stress sweep plot with storage modulus G′ and loss modulus G″ (b), both for the carbon ink used for the printing of the monoliths.

The rheological properties of the as‐prepared carbon ink were analyzed prior to printing to ensure good printing results. To achieve continuous extrusion and stable structures after extrusion, the ink must have a high viscosity at low shear rates and show shear‐thinning behavior so that it starts to flow when applying a certain stress and remains in its shape when the pressure is released [27, 28, 29]. The flow curve and the strain sweep of the ink can be found in Figure 1. The viscosity in the flow curve decays linearly on the log‐log plot with increasing shear rate, and the carbon ink exhibits a strong shear‐thinning behavior. This scaling relationship is commonly observed and called either the power law model or the Ostwald‐de Waele relationship [30], and is used to describe the flow curves of non‐Newtonian fluids. For the ink here, the corresponding value for the power‐law coefficient is 0.26. This value is smaller than one, which is characteristic for shear thinning fluids. The consistency had a value of 5.4∙105 Pa s^0.26^. In the controlled strain sweep plot, the storage (G′) and loss (G″) moduli are constant at a low shear stress, where the ink behaves elastically. This demonstrates that the ink can hold its shape after extrusion. With increasing shear stress, the behavior of the ink changes from elastic to viscous. Both G′ and G″ moduli start to drop, indicating yielding and transition to a liquid‐like behavior. At a certain point, the G′ and G″ moduli cross‐over. This point is called the yield point and has here a value of 150 Pa. Beyond the yield point, the ink starts to flow, which is desirable for a continuous and homogeneous extrusion of the ink. At an even higher shear stress, the G′ modulus decreases rapidly due to the breakdown of the internal structure of the ink.

**FIGURE 2:**
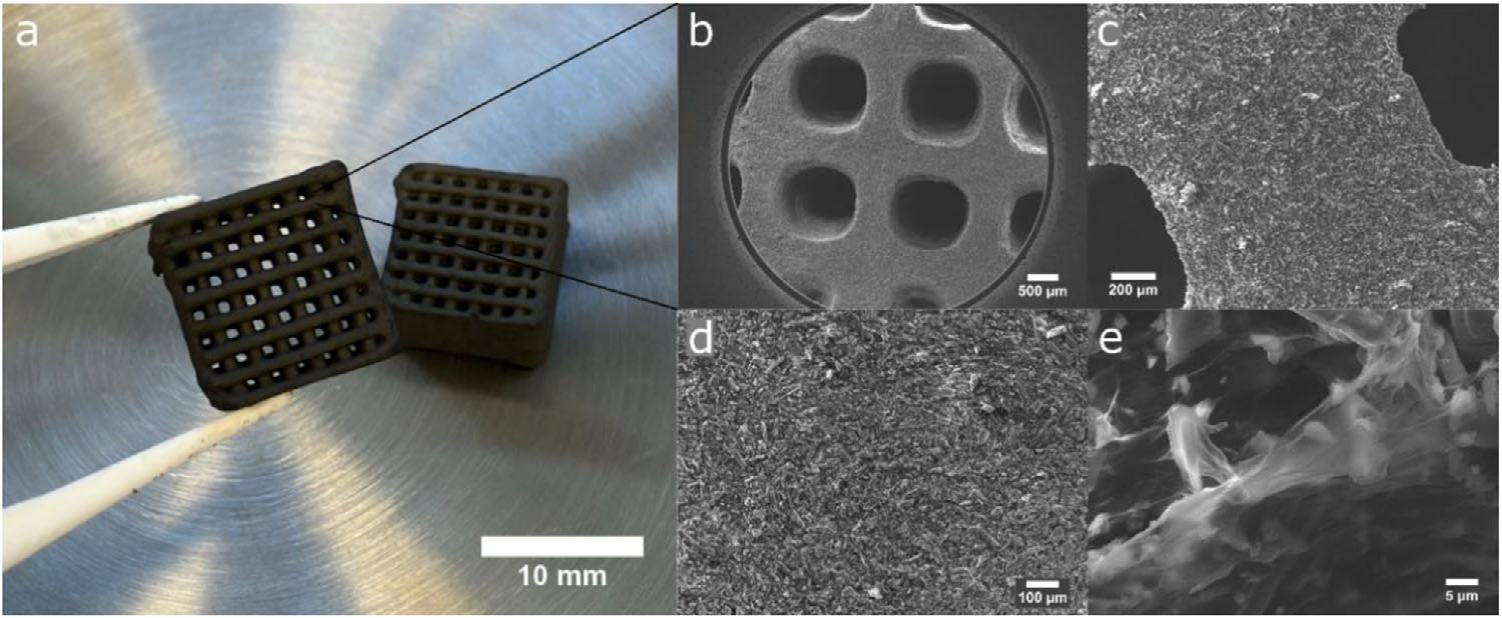
Photograph (a) and SEM results of CM (b–e).

Taking this all together, analyses of both the flow curve and the controlled stress sweep show that the carbon ink exhibits the desirable properties for its processing via direct ink writing, which is further supported by the feature of the photograph of the printed monoliths CM shown in Figure 2a. The microstructure of the monoliths was analyzed by scanning electron microscopy (SEM). SEM images of the monoliths after freeze‐drying are presented in Figure 2b–e. It can be seen that 3D printing gives monoliths with geometrically defined and regular grid structures with sharp edges. The texture of the monoliths is homogeneous. The monoliths are porous and have a typical cryogel scaffold. The activated carbon particles are connected by the graphene oxide (GO) sheets and alginate binder, resulting in a well‐intergrown structure with pores, allowing for gas diffusion through the structure.

### 2.2 Amine‐Functionalization of Monoliths

The CMs were functionalized with APTES to provide chemisorption sites to increase the affinity to CO_2_ [12, 13]. The nitrogen content before and after the modification reaction was quantified by combustion analysis (Table S2), as the difference in the nitrogen content is correlated with the content of amino groups of the APTES‐derived species in ACM. After modification, the nitrogen content of the modified monolith (ACM) was increased from 0.4  to 3.1 mmol g^−1^. For the unmodified monolith (CM), nitrogen was present in a very small proportion in the form of functional groups in activated carbon particles and GO platelets, while in ACM, the majority of nitrogen was in the form of amino groups of the APTES‐derived species. The amine content of 2.7 mmol g^−1^ in the bulk material corresponds to the difference between the inherent nitrogen content of CM and the total nitrogen content of ACM. This is comparable to the content of the APTES‐derived species determined in functionalized silica in previous studies [31, 32, 33, 34]. The amine content of 2.7 mmol g^−1^ corresponds to an average amine surface density of 2.2 N atoms nm^−2^.

**FIGURE 3:**
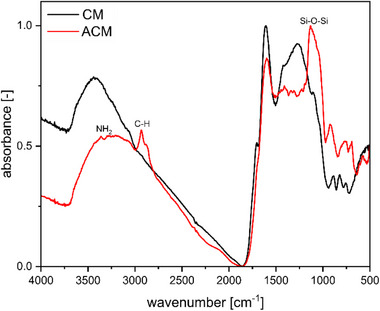
IR spectra of CM and ACM.

The presence of APTES‐derived species and their cross‐linked silane networks in ACM was confirmed by IR analysis, and corresponding spectra for CM and ACM are shown in Figure 3.

Both spectra show the presence of various bands in the range of 750–1750 cm^−1^, which were assigned to various vibrations from the carbon scaffold of AC, GO, and Alg and their functional oxygen groups, and in the spectrum of ACM also from APTES‐derived species and their cross‐linked silane networks (Table S3) [35, 36, 37]. The broad band at 3000–3500 cm^−1^ was assigned to vibrations of O─H groups present in the form of carboxyl and hydroxyl groups in the carbon monolith [35] and silanol groups in hydrolyzed APTES [37]. The IR spectrum of ACM shows additional bands, which indicate the presence of APTES‐derived species and their cross‐linked silane networks. Asymmetric and symmetric stretching bands assigned to Si─O─Si are visible between 1000 and 1200 cm^−1^ and at 800 cm^−1^, respectively [37]. Furthermore, C─H stretching bands of the propyl chain appear clearly at 2880 and 2930 cm^−1^ and tiny but very typical N─H stretching bands at 3290 and 3360 cm^−1^ [37, 38]. While the N─H stretching bands overlap with the broad O─H stretching band and are therefore subtle, the presence of APTES‐derived species and their amino groups is further confirmed by the combination of the additional IR features and elemental analysis. These combined findings provide strong evidence for the successful grafting of APTES onto the carbon monolith.

**FIGURE 4:**
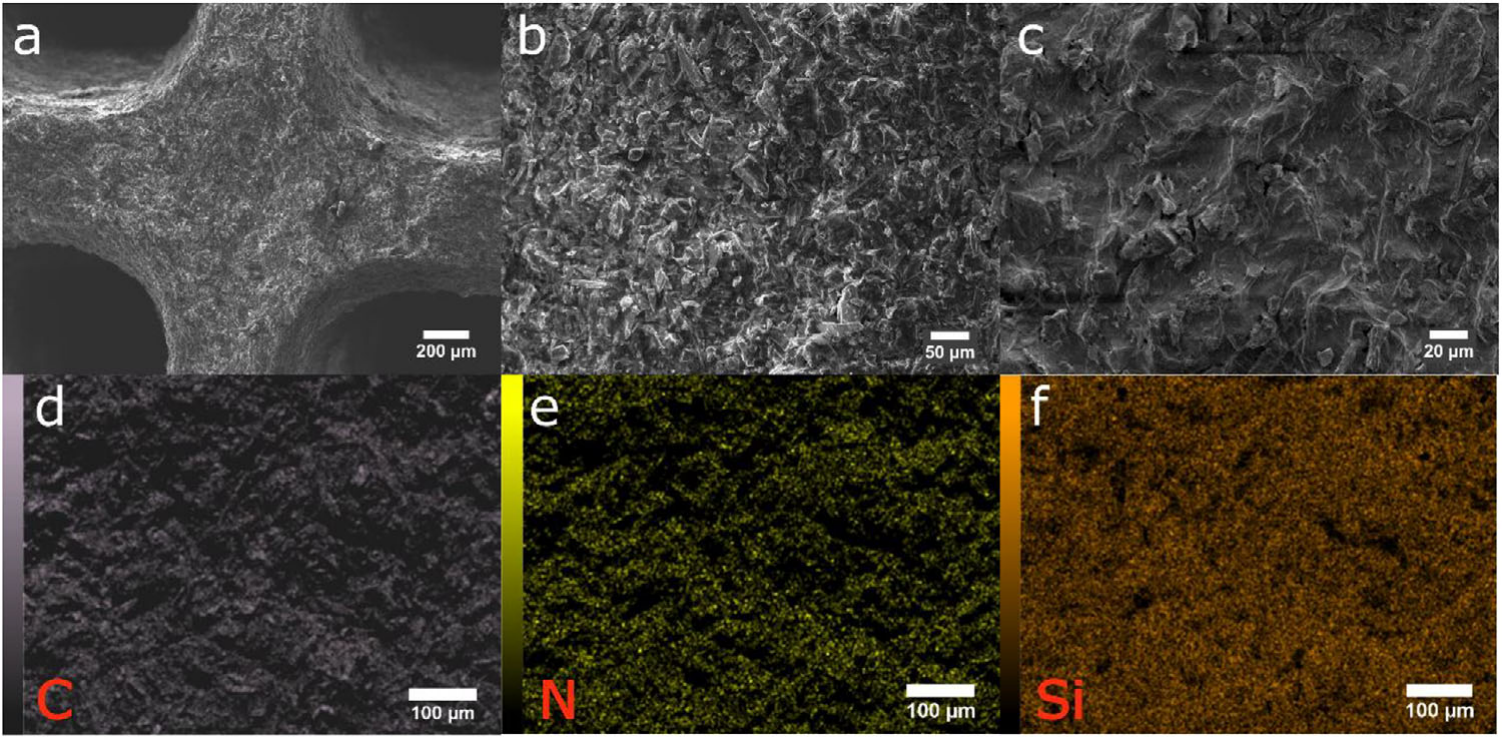
SEM results of ACM (a–c) and elemental mapping of C, N, and Si obtained with EDS on ACM (d–f).

The influence of the cross‐polymerization of APTES on the microstructure was investigated by SEM. The images in Figure 4a–c shows that the cross‐polymerization of APTES across the surface of the CM causes only minor changes in the texture of the ACM monoliths, giving them slightly smoother outer surfaces than the CM monoliths. Additionally, an elemental mapping of nitrogen was conducted with EDS to examine the distribution of APTES‐derived surface layer and its cross‐linked silane networks. EDS spectra of the surface of CM and ACM are displayed in Figure S1, showing a significantly higher amount of N and Si in ACM than CM, confirming the successful grafting of APTES and the formation of aminosilane species across the ACM surface. The homogeneous distribution of C, N, and Si elements in ACM in Figure 4d–f indicates that the APTES‐derived layer is uniformly formed and cross‐linked on the surface. Minor intensity variations are attributed to surface texture and roughness.

**FIGURE 5:**
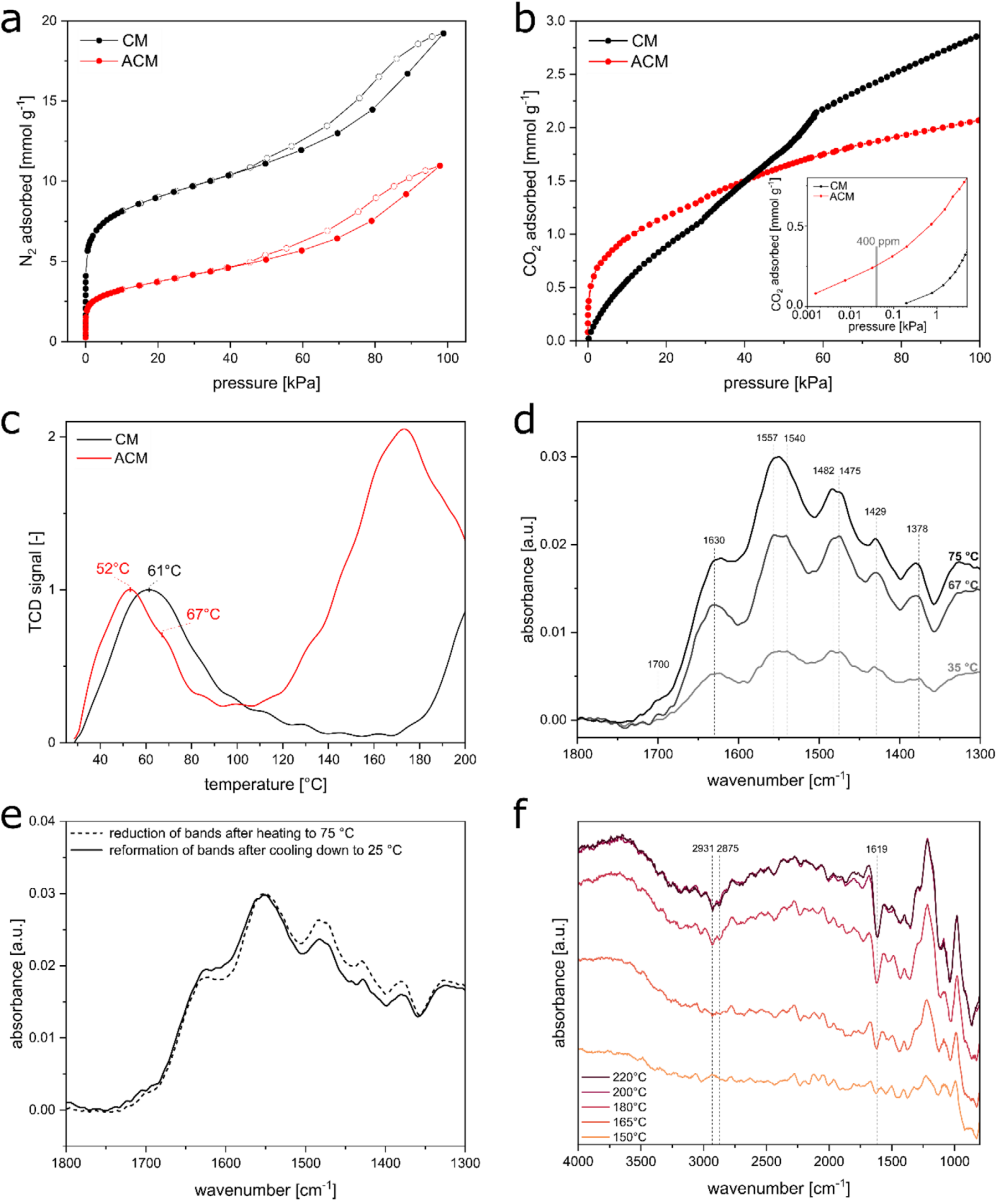
N_2_ adsorption–desorption isotherms at 77 K (a), CO_2_ adsorption isotherms at 278 K (b), temperature‐programmed desorption of CO_2_ under N_2_ atmosphere on CM and ACM (c), difference IR spectra of ACM at 35°C, 67°C, and 75°C (d), difference IR spectra of ACM between 75°C and 25°C before heating to 75°C and after cooling down (e) and difference IR spectrum of ACM at 150°C, 165°C, 180°C, 200°C, and 220°C (f).

The impact of the cross‐polymerization of APTES across the surface on the porosity of ACM was examined using N_2_ adsorption–desorption measurements, as shown in Figure 5a. Both CM and ACM exhibit N_2_ isotherms with steep initial parts and hysteresis loops characteristic of micro‐ and mesoporous materials. After the modification with APTES, a significant reduction in N_2_ uptake is observed. This is reflected in a decrease in BET surface area from 740 m^2^ g^−1^ of CM to 310 m^2^ g^−1^ of ACM, and in pore volume from 0.67 to 0.38 cm^3^ g^−1^ (Table S4). Analysis of the t‐plot (Figure S2) indicates that this reduction arises from both micropores and meso‐/macropores. Specifically, the micropore area decreases from 340 to 70 m^2^ g^−1^, and micropore volume from 0.14 to 0.023 cm^3^ g^−1^, showing that approx. 80% of the micropores become inaccessible after modification. This loss is likely due to pore blocking by APTES‐derived species and their cross‐linked silane networks. In contrast, the volume of meso‐ and macropores is reduced by approx. 30%, presumably due to the grafting and cross‐linking of APTES‐derived silanes within the pores. Additionally, blocking of mesopores by the APTES‐derived layer could occur, as it has been previously reported for mesoporous silica modified with APTES in the presence of water, resulting in reduced surface area and porosity [32, 39]. The t‐plots and a table of all textural properties can be found in the SI.

### 2.3 CO_2_ Adsorption

CO_2_ adsorption isotherms of CM and ACM at 278 K are presented in Figure 5b. ACM exhibits higher CO_2_ adsorption than CM at partial pressures below 45 kPa, but lower CO_2_ adsorption at pressures above 45 kPa. At 278 K and pressures below 10 kPa, CO_2_ adsorption on CM increases nearly linearly, whereas ACM shows a steep initial uptake in the low‐pressure region with a subsequent flatter increase (CO_2_ adsorption isotherms of ACM at 288 and 298 K are presented in Figure S3). This difference in slope at low pressures is attributed to a higher heat of adsorption in ACM, consistent with CO_2_ chemisorption on ACM due to chemical interactions between the amino groups and CO_2_. Primary amino groups are known to form chemical bonds with CO_2_ molecules, enhancing both CO_2_ uptake and selectivity at low partial pressures [40]. The reduced CO_2_ adsorption at higher pressures originates presumably from the decreased surface area and pore volume after the amination of CM. At elevated pressure, physisorption becomes more dominant, and the reduced porosity leads to a decrease in adsorptivity [40, 41]. A first‐order assessment of a sorbent's suitability for DAC can be made by evaluating its equilibrium CO_2_ adsorption capacity at a pressure of 0.04 kPa (400 ppm) and temperatures relevant for ambient air capture of CO_2_ [42]. Under these conditions, CM adsorbs less than 0.01 mmol g^−1^ CO_2_, while ACM achieves 0.25 mmol g^−1^ (Table S5). This significant difference demonstrates that amination with APTES enhances CO_2_ adsorption under DAC‐relevant conditions.

As highlighted in the introduction, not only a high CO_2_ adsorption capacity but also a low energy demand for the regeneration of the sorbent is essential for high‐performance sorbent materials. This energy demand is characterized by the heat capacity and the enthalpy of adsorption and desorption, respectively of the sorbent material [42, 43]. A low desorption enthalpy results in a lower desorption temperature and, consequently, reduced energy demand. Temperature‐programmed desorption (TPD) was used to determine the CO_2_ desorption temperature, with the corresponding TPD curves shown in Figure 5c. For ACM, the TPD curve displays two overlapping peaks between 30°C and 75°C, and a second peak with an onset at 125°C. The overlapping peaks are attributed to the desorption of physisorbed and chemisorbed CO_2_. The presence and desorption of chemisorbed CO_2_ was confirmed via IR spectroscopy. Absorbance spectra were recorded at different temperatures with the single beam spectrum without the sample obtained at 25°C serving as the background spectrum for all subsequent spectra (Figure S4). To highlight temperature‐dependent spectral changes, difference spectra were generated by subtracting the absorbance spectra recorded at elevated temperatures from the reference spectrum at 25°C (Figure S5). This approach allows for the identification of spectral features corresponding to species present at 25°C but absent at higher temperatures, thereby indicating thermally induced desorption. The resulting difference spectra are presented in Figure 5d. The spectra reveal characteristic bands of CO_2_ adsorbed as ammonium carbamate. Bands at 1378 and 1430 cm^−1^ correspond to symmetric stretching vibrations of COO^−^ and the band at 1482 cm^−1^ is assigned to symmetric deformation vibration of NH_3_^+^. The asymmetric stretching band of COO^−^ appears between 1540 and 1557 cm^−1^, and the asymmetric deformation band of NH_3_^+^ is observed at 1630 cm^−1^ [44, 45, 46]. The IR spectroscopic experiments clearly confirm that CO_2_ is adsorbed as ammonium carbamate on ACM from ambient air. Desorption of chemisorbed CO_2_ begins immediately upon heating, with a decrease in the intensity of ammonium carbamate bands already visible at 35°C. The intensity of these bands continues to decline with increasing temperature across the range of the two overlapping TPD peaks, confirming their attribution to CO_2_ desorption. After heating to 75°C, the sample was cooled in air to 25°C over 20 min, and a subsequent IR spectrum was recorded (Figure S6). Figure 5e shows the differences between these spectra at 25°C before heating and after cooling down while exposed to air, and the spectrum at 75°C. The ammonium carbamate bands return to their original intensity upon cooling while exposed to air, indicating that CO_2_ chemisorption on ACM from ambient air is fast and reversible. This reversibility makes ACM a suitable sorbent for cyclic TSA processes. IR spectroscopy was also employed to investigate the origin of the second TPD peak of ACM, which appears with a maximum at 175°C. Figure 5f displays the spectral differences above 150°C relative to the spectrum at 105°C, showing changes between 150°C and 200°C. No further spectral changes are observed above 200°C. The decreasing bands at 2931, 2978, and 1619 cm^−1^, assigned to CH_2_ stretching and NH_2_ deformation vibrations [45], suggest thermal degradation or volatilization of the aminosilane in this temperature range, which accounts for the second TPD peak. The absence of a corresponding peak in the TPD curve of CM supports this interpretation. The peak with an onset at 175°C in the TPD curve of CM may result from CO_2_ release due to decomposition of carboxylic acid and other oxygen‐containing functional groups [47]. Although CO_2_ desorption occurs in a similar temperature range for both materials, the peak for CM is slightly shifted to higher temperatures. This shift may be due to the higher microporosity of CM, which can lead to elevated apparent desorption temperatures due to kinetic limitations [48] and strong electrostatic interactions within the micropores [49].

In short, TPD analysis demonstrates that complete CO_2_ desorption can be achieved at temperatures above 75°C. The aminosilane‐modified carbon monolith not only exhibits significant CO_2_ adsorption capacity at atmospheric concentrations but can also be regenerated at relatively low temperatures, highlighting its potential as an energy‐efficient sorbent for DAC applications. To note, the regeneration temperature was determined under N_2_ purge to isolate thermal effects, which provides a benchmark for the performance of the material rather than a full DAC process. To provide a context for these results, a TPD measurement with the commercial DAC chemisorbent Lewatit VP OC 1065 was conducted under identical conditions. The TPD curve in Figure S7 shows CO_2_ desorption between 30°C and 95°C with a peak at 75°C. ACM exhibits CO_2_ desorption at a lower temperature, indicating improved regeneration behavior compared to Lewatit VP OC 1065. Future work will be needed to evaluate the regeneration behavior of ACM under realistic DAC conditions. Finally, our study on the CO_2_ adsorption kinetics of ACM obtained by thermogravimetric analysis after pre‑degassing at 353 K (Figure S8) validates that the grafting of APTES, although possibly reducing accessibility to micropores, does not hinder CO_2_ transport to the active sites. In fact, the kinetics remain sufficiently fast for practical DAC applications.

## 3 Conclusion

In this study, monolith structures with activated carbon as a major component were prepared by direct ink writing, and subsequently functionalized with APTES. The resulting aminated monoliths exhibited a CO_2_ adsorption capacity more than 25 times higher than that of the unmodified monoliths under ambient conditions. Although some amine‑impregnated carbon sorbents show higher equilibrium CO_2_ uptakes under DAC conditions (Table S1), their performance is often limited by amine loss and degradation during cycling. In contrast, the covalently grafted APTES layer in our monolithic sorbent provides enhanced stability, and the use of activated carbon as a low‑cost, scalable substrate further supports its practical applicability for DAC.

Reversible adsorption of CO_2_ as ammonium carbamate was demonstrated, with desorption occurring within a temperature range of 35°C–75°C. The central hypothesis of this work posits that effective DAC systems require sorbent materials that are not only chemically tailored for high CO_2_ affinity but also structurally optimized to enable rapid mass and heat transfer, as well as potential heat integration. Furthermore, sufficient thermal conductivity of the sorbent is essential to facilitate efficient TSA processes, thereby minimizing both energy consumption and the spatial footprint of DAC devices. To the best of our knowledge, this study provides the first documented implementation of 3D printing for the fabrication of aminated activated carbon monoliths specifically designed for DAC applications. This approach enables the customization of monolith geometries to suit various reactor configurations, offering a versatile platform for process optimization. The relatively low desorption temperature of 75 °C supports energy‐efficient regeneration of the sorbent, further contributing to the reduction of operational energy demand. Additionally, carbon‐based sorbents present a cost‐effective alternative due to their natural abundance and inherently high thermal conductivity. Humidity is known to enhance CO_2_ adsorption for many amine‑functionalized sorbents by promoting water‑assisted adsorption pathways and increasing amine efficiency, although water co‑adsorption can also increase regeneration energy demand. Future research will focus on evaluating the influence of ambient humidity on CO_2_ adsorption and assessing the long‐term stability of amine‐functionalized carbon sorbents under cyclic operating conditions to further validate their applicability in DAC systems.

## 4 Experimental Section/Methods

### 4.1 Materials

Sodium alginate (NaAlg, from Alfa Aesar), calcium chloride (97%, Alfa Aesar), and 3‐aminopropyltriethoxysilane (MQ200, Sigma–Aldrich) (APTES) were purchased and used without further purification or pre‐treatment. Activated carbon PAK A 1120 H (AC) was purchased from CarboTech GmbH, and a 1 wt.‐% dispersion of graphene oxide (GO) in water from Global Graphene Group. Lewatit VP OC 1065 was purchased from Lanxess.

### 4.2 Preparation of Carbon Inks

For the preparation of carbon ink, 1.5 g of an aqueous solution of NaAlg at a concentration of 80 g L^−1^ was added to 20 mL of GO dispersion (1 wt.‐%). The mixture was thoroughly stirred until a homogeneous viscous dispersion was obtained. Afterward, 2.4 g of AC was added under constant stirring, resulting in a homogeneous black paste.

### 4.3 Direct Ink Writing

For the printing of monolith structures, the direct ink writing technique was applied on a custom‐made setup. An Ultimaker 2+ Connect 3D printer was coupled with a pressure‐controlled fluid dispenser from Nordson EFD. The cartridge of the dispenser was mounted on the print head for the extrusion of the material. Prior to printing, the paste was extruded through a 400 µm nozzle to avoid nozzle clogging during the printing. In the printing process, the material was extruded through a 400 µm nozzle under a dispensing pressure of 0.27–0.35 bar. The printing rate was set to 30 mm s^−1^, and a layer height of 400 µm was set. Square grid structures with a size of 12 mm ×  12 mm and a height of 10 mm were printed. After printing, the monolith structure was wetted with 5 m CaCl_2_ solution for 3 min and subsequently immersed in a 0.1 m CaCl_2_ solution for 3 h to cross‐link the alginate binder. Afterward, the monolith was washed with distilled water to remove excess CaCl_2_ and then freeze‐dried, giving a carbon monolith (CM).

### 4.4 Amine‐Modification

The freeze‐dried monolith was immersed in a freshly prepared solution of APTES (1 mL, 4.3 mmol) in distilled water (50 mL). The mixture was gently mixed with a rotary shaker at a shaking speed of 100 rpm. After 20 h, the monolith was separated from the solution, washed with distilled water, and dried at 65°C for 24 h. An amine‐modified carbon monolith (ACM) was obtained.

### 4.5 Characterization

The flow properties of the carbon ink were analyzed with a MCR 301 rheometer from Anton Paar. The viscosity was measured in dependence of shear rate with a logarithmic ramp profile in the range of 10^−3^–10^3^ s^−1^, and a strain sweep was recorded with a logarithmic ramp profile from 10^−1^ to 10^3^ s^−1^. Infrared spectra of CM and ACM were recorded in the range of 400–4000 cm^−1^ with an Alpha II with diffuse reflectance infrared Fourier transform spectroscopy (DRIFTS) setup from Bruker. The nitrogen content of CM and ACM was determined by combustion analysis by MikroLab Kolbe. Adsorption and desorption isotherms were collected with a Vapor 200 C from 3P Instruments GmbH & Co. KG. N_2_ adsorption and desorption isotherms of CM and ACM were collected at 77 K, and CO_2_ adsorption isotherms at 278 K. Prior to the measurements, the samples were degassed at 353 K for 15 h. A JEOL JSM‐IT 800 scanning electron microscope (SEM) was used to examine the microstructure of the monoliths. An elemental mapping with energy‐dispersive spectroscopy (EDS) was conducted with the same instrument. The desorption temperature of CM, ACM, and Lewatit VP OC 1065 for CO_2_ was determined by temperature‐programmed desorption (TPD). Prior to the measurement, the samples were degassed at 353 K. Then, the CO_2_ flow was started to saturate the samples with CO_2_. For the measurement, N_2_ flow was started, and a temperature ramp at 5 K min^−1^ from 303 to 473 K was applied. Desorption of CO_2_ and release of decomposition products were detected with a thermal conductivity detector (TCD). The measurements were conducted on an AMI‐400 Chemisorption Analyzer from Altamira Instruments. The TPD curves were normalized to the maximum of the desorption peaks. Desorption of CO_2_ was further studied on ACM with infrared spectroscopy on a Varian 610‐IR microscope coupled to a Varian 670‐IR spectrometer. Spectra were recorded in reflection mode in the range of 650–4000 cm^−1^. A Linkam THMS 600 heating stage was used to measure spectra at elevated temperatures. The sample was grinded and a thin film was placed on aluminum foil, which was then placed on the heating stage. Aluminum foil was chosen because it is IR reflective and provides high heat conduction. The sample was kept in ambient air prior to the measurement to allow adsorption of CO_2_.

## Conflicts of Interest

The authors declare that the material described in this manuscript and its synthetic route are the subject of a pending patent application filed by Volkswagen AG.

## Supporting information




**Supporting File**: gch270105‐sup‐0001‐SuppMat.pdf.

## Data Availability

The data that support the findings of this study are available from the corresponding author upon reasonable request.
