# Supplementary material for: Amine‐Functionalized Activated Carbon Monoliths by 3D Printing for Direct Air Capture
Source: Glob Chall. 2026 Apr 10;10(4):e70105. doi: 10.1002/gch2.70105 (PMC13066914; doi:10.1002/gch2.70105)
Supplement: Supplementary file 1 — Supporting File: gch270105‐sup‐0001‐SuppMat.pdf. [file GCH2-10-e70105-s001.pdf]

Supporting Information

Amine-functionalized Activated Carbon Monoliths by 3D printing for Direct Air Capture

*Johanna Fricke, Zoltán Bacsik, Christine Schütz, Marc Rüggeberg, Anju Pal, Niklas Hedin, Jiayin Yuan**

Table S1. Amine-functionalized carbon materials for CO_2 adsorption under DAC conditions.

Carbon material	Modification	Amine	CO_2 uptake [mmol g^{-1}]	CO_2 conc.	Temp.	Ref.
Mesoporous carbon	impregnation	PEI	2.25	400 ppm (dry)	25 °C	[1]
Mesoporous carbon	impregnation	PEI	2.65	400 ppm (dry)	40 °C	[2]
Activated carbon paper	impregnation	PEI	0.46	400 ppm (dry)	20 °C	[3]
Reduced graphene oxide	impregnation	PEI	0.61	400 ppm (dry)	20 °C	[4]
Carbon fibers	impregnation	PEI	0.45	400 ppm (dry)	25 °C	[5]
Activated carbon fiber cloth	Vapor-phase funct.	azasilane	0.083	400 ppm (dry)		[6]
Thermally annealed graphite oxide	impregnation	PEI	~0.9	400 ppm (dry)	25 °C	[7]
**Activated carbon**	**grafting**	**APTES**	**0.25**	**400 ppm (dry)**	**5 °C**	**This work**

Table S2. Nitrogen content in carbon monolith CM and amine-functionalized carbon monolith ACM determined with elemental analysis by double-determination.

	N [wt%]	N [mmol g^{-1}]
CM	**0.56 ± 0.01**	**0.4 ± 0.1**
ACM	**4.38 ± 0.01**	**3.1 ± 0.1**

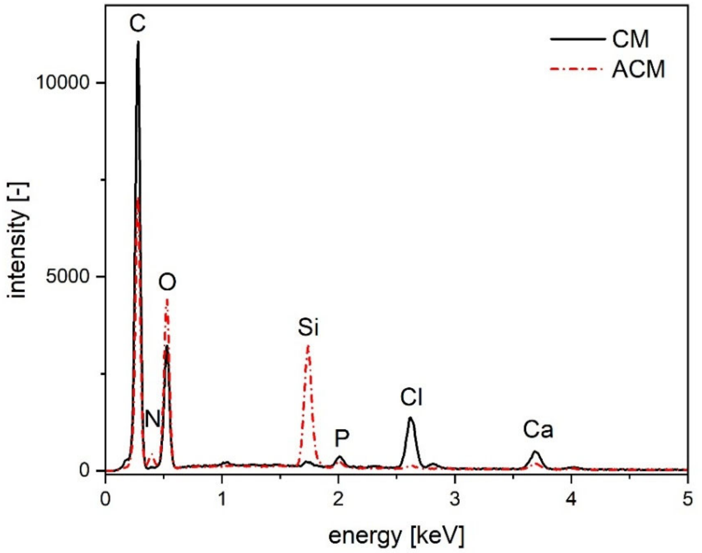

Figure S1. Energy-dispersive spectra of CM and ACM.

Table S3. IR bands of CM and ACM in Figure 3.

	CM	ACM
O-H stretching bands	**3000 – 3500 cm^{-1}**	**3000 – 3500 cm^{-1}**
N-H stretching bands	**-**	**3290 cm^{-1}, 3360 cm^{-1}**
C-H stretching bands	**-**	**2880 cm^{-1}, 2930 cm^{-1}**
Si-O-Si stretching bands	**-**	**1000 – 1200 cm^{-1}, 800 cm^{-1}**
Carbon scaffold and functional oxygen groups of AC, GO and Alg	**750 – 1750 cm^{-1}**	**750 – 1750 cm^{-1}**

Table S4. BET surface area and pore volume, and external surface area, micropore area and micropore volume determined from the t-plot of CM and ACM.

	BET surface area [m^2 g^{-1}]	Pore volume [cm^3 g^{-1}]	External surface area [m^2 g^{-1}]	Micropore area [m^2 g^{-1}]	Micropore volume [cm^3 g^{-1}]
CM	**740 ± 10**	**0.67 ± 0.04**	**400 ± 10**	**340 ± 10**	**0.14 ± 0.01**
ACM	**310 ± 10**	**0.38 ± 0.03**	**240 ± 10**	**70 ± 10**	**0.023 ± 0.01**

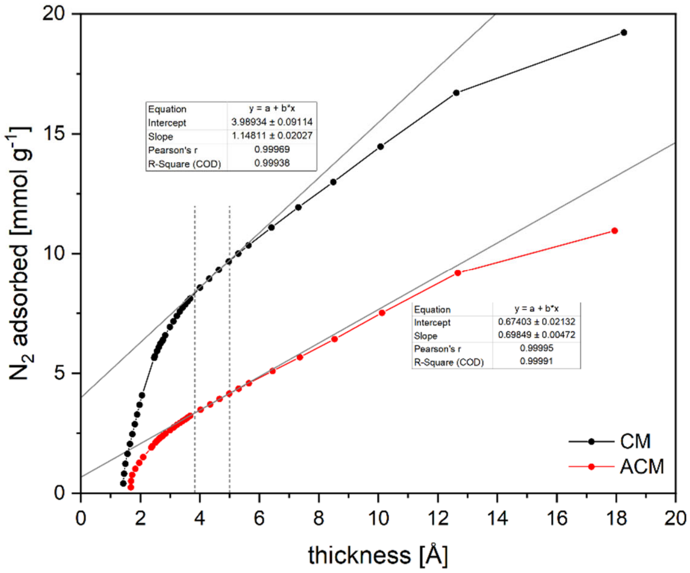

Figure S2. t-plots of N_2 adsorption isotherms of CM and ACM at 77 K. Thickness values t were calculated with Harkins-Jura equation and linear regression was conducted in the range of 3.8 Å to 5 Å.

Table S5. CO_2 adsorption capacity at 278 K determined from adsorption isotherms and CO_2 desorption temperature range of CM and ACM determined from TPD.

	CO_2 adsorption capacity	CO_2 desorption temperature range
CM	**< 0.01 mmol g^{-1}**	**30 °C – 90 °C**
ACM	**0.25 mmol g^{-1}**	**30 °C – 75 °C**

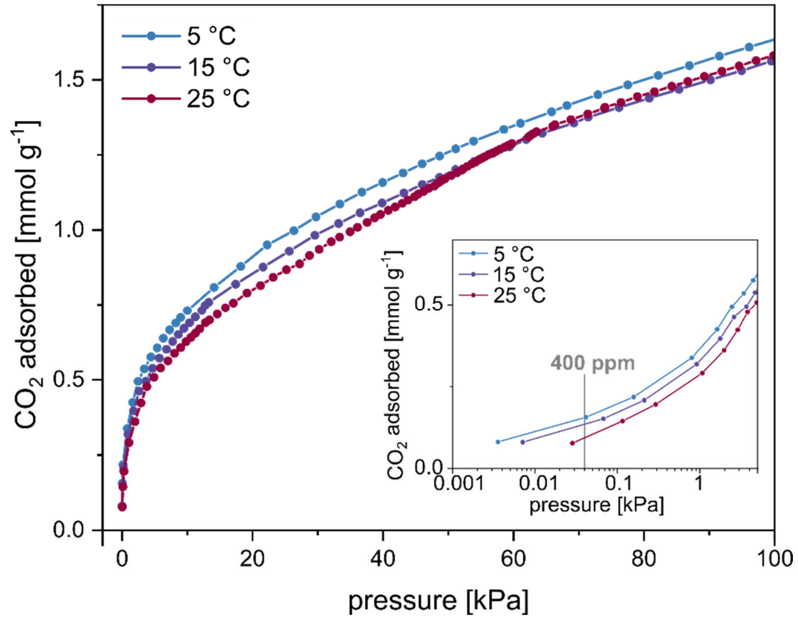

Figure S3. CO_2 adsorption isotherms of ACM at 5, 15 and 25 °C.

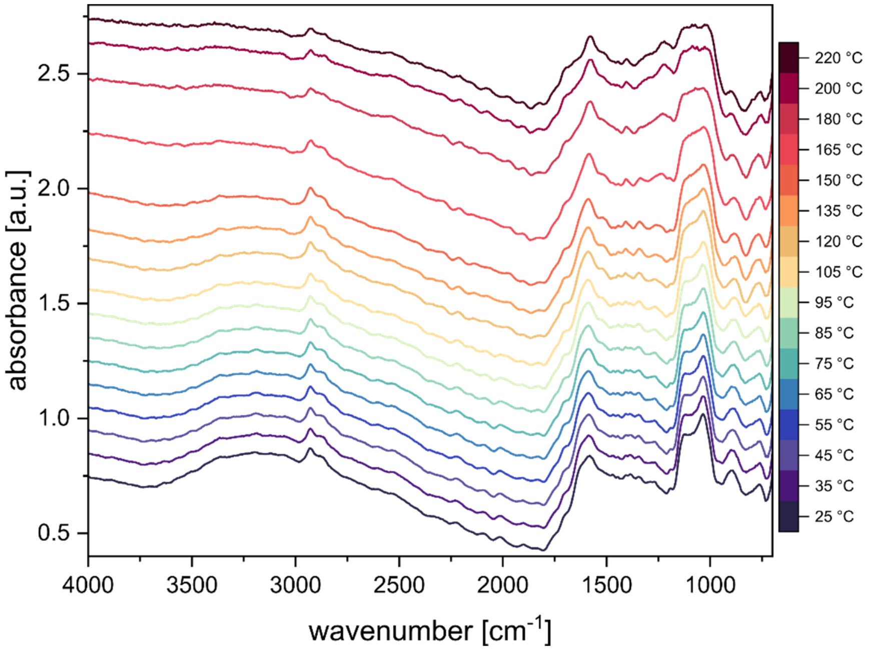

Figure S4. IR spectra of ACM at different temperatures.

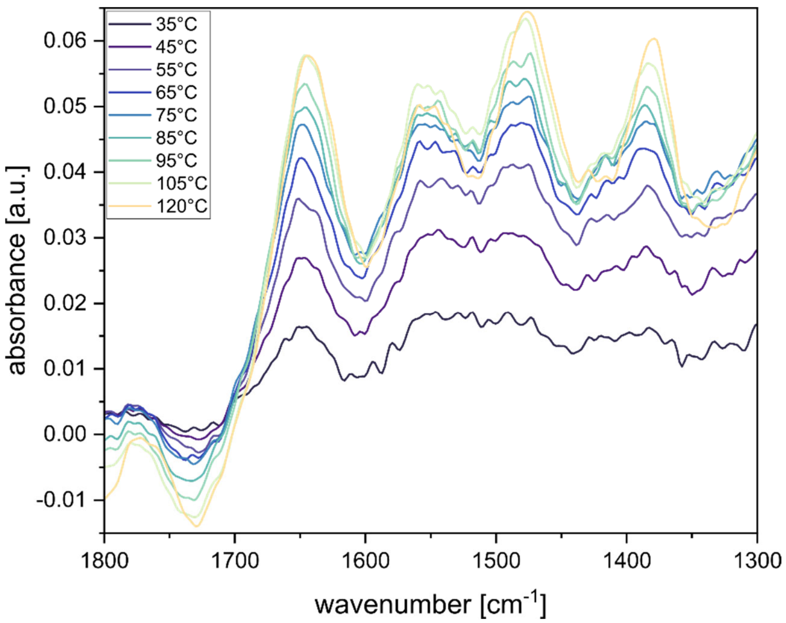

Figure S5. Difference IR spectra of ACM between elevated temperatures and 25 °C.

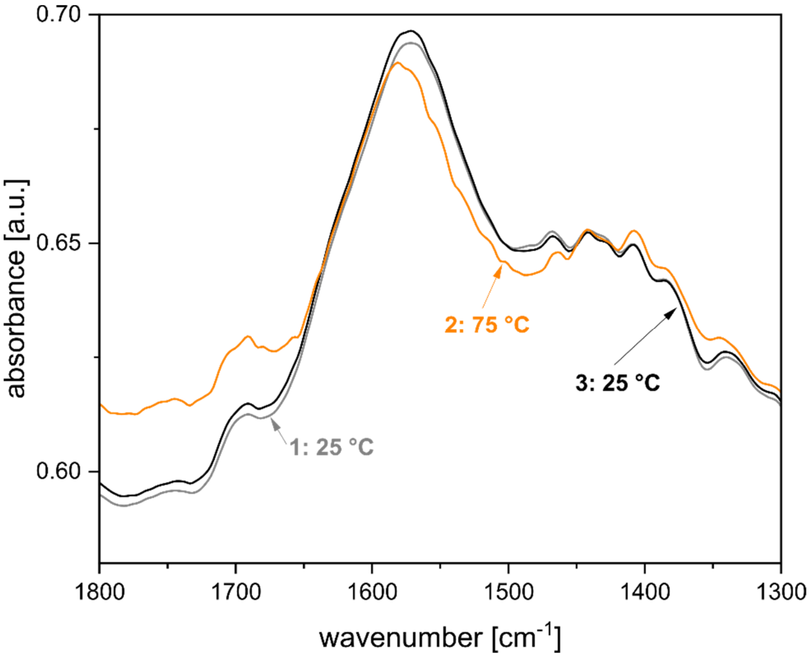

Figure S6. IR Spectra at 25 °C before heating, at 75 °C, and after cooling down to 25 °C.

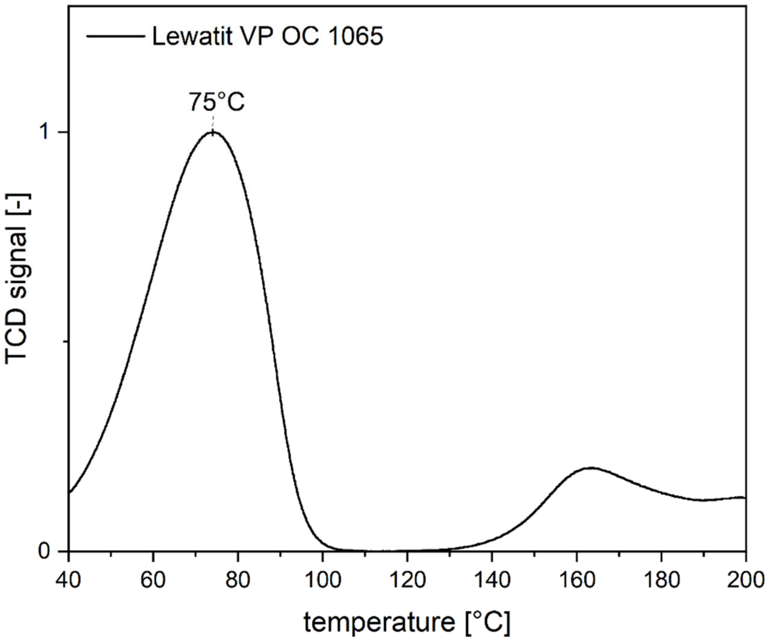

Figure S7. CO_2 TPD curve of Lewatit VP OC 1065.

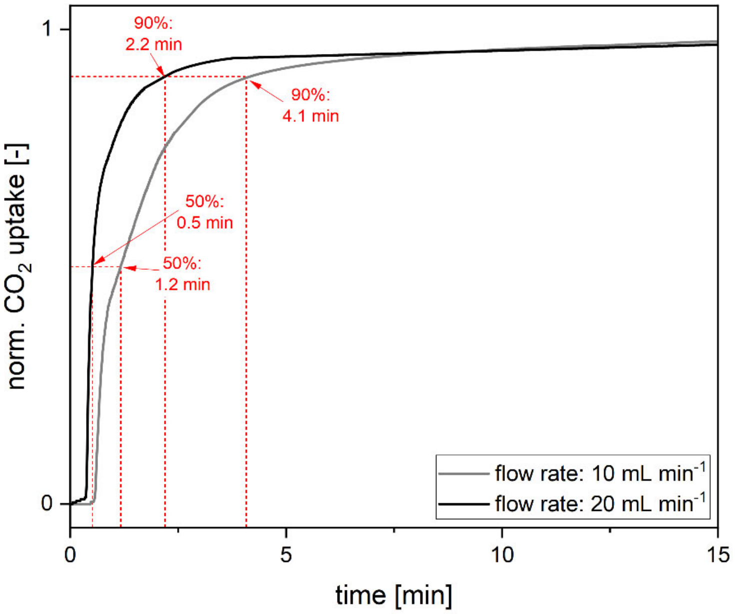

Figure S8. Kinetics of CO_2 adsorption of ACM experimentally determined with thermogravimetric analysis after degassing and at CO_2 flow rates of 10 and 20 mL min^{-1}.

Figure S8 shows the CO_2 adsorption kinetics of ACM obtained by thermogravimetric analysis after pre-degassing at 353 K. The curves demonstrate a fast initial uptake of CO_2, with 50 %

saturation reached within 0.5 and 1.2 min and 90 % saturation within 2.2 and 4.1 min depending on the flow rate. The rapid rise in mass confirms that, despite the reduction in microporosity after amination, the material maintains fast adsorption kinetics under dynamic flow conditions. The stabilized plateau region indicates that equilibrium is reached within a few minutes, supporting the suitability of ACM for DAC processes in which rapid cycling is required.

These results validate that the grafting of APTES, although reducing the accessible micropore volume, does not significantly hinder CO_2 transport to the active sites. Instead, the kinetics remain sufficiently fast for practical DAC applications, where short adsorption-desorption cycles are essential. The results complement the qualitative kinetic in situ IR study in the main text (Figure 5e).

References

1. Wang, J. *et al.* Direct Capture of Low-Concentration CO_2 on Mesoporous Carbon-Supported Solid Amine Adsorbents at Ambient Temperature. *Ind. Eng. Chem. Res.* **54**, 5319–5327 (2015).
2. Yihang, L. *et al.* Pore-modified mesoporous-carbon-based supported amine adsorbents for direct air capture: Adsorption performance and concerted reaction mechanism. *Separation and Purification Technology* **378**, 134535 (2025).
3. Ai, J. *et al.* Highly aminated carbon paper for CO_2 capture. *Sustainable Chemistry for Climate Action* **7**, 100149 (2025).
4. Ai, J. *et al.* Polyethyleneimine-functionalized graphene oxide aerogels for direct air capture. *Chemical Engineering Journal* **506**, 159963 (2025).
5. Lee, W. H. *et al.* Sorbent-coated carbon fibers for direct air capture using electrically driven temperature swing adsorption. *Joule* **7**, 1241–1259 (2023).
6. Prünte, S. *et al.* Surface functionalization of microporous carbon fibers by vapor phase methods for CO_2 capture. *Journal of Vacuum Science & Technology A* **41**, 032403 (2023).
7. Song, M., Kim, J., Jones, C. W. & Lively, R. P. Electrically Conductive Amine Functionalized Reduced Graphite Oxide Foam for CO_2 Removal from the Air. *ACS Appl. Mater. Interfaces* **17**, 65565–65576 (2025).
